# Evaluation of several strategies for controlling canker plant disease caused by *Pseudomonas syringae*

**DOI:** 10.22099/mbrc.2024.51122.2034

**Published:** 2025

**Authors:** Reyhaneh Ravanbakhshian-HabibAbadi, Mandana Behbahani, Hassan Mohabatkar

**Affiliations:** Department of Biotechnology, Faculty of Biological Science and Technology, University of Isfahan

**Keywords:** Biological agents, Secondary metabolites, Nanoparticles, Bacteriophages, Antimicrobial peptides

## Abstract

*Pseudomonas syringae* is a gram-negative bacterium that causes a diversity of diseases in numerous plants. Strategies to inhibit *P. syringae* growth include protective procedures; however, controlling the disease is complicated due to its rapid spread. Several antimicrobial agents can prevent this disease, such as chemical compounds, biological agents, secondary metabolites, nanoparticles, bacteriophages, and antimicrobial peptides (AMPs). The most effective way to control the disease is through chemical control. Using copper compounds and antibiotics is a conventional practice to decrease canker disease symptoms. However, due to environmental pollution caused by chemicals and bactericides and the resistance of different pathovars of *P. syringae*, other methods for bacterial pathogens control are needed. Biological control, using antagonistic bacteria has shown promising results against *P. syringae* under in vitro conditions. New studies focus on using secondary metabolites from plants to control plant diseases. Studies have shown that essential oils when preserved from degradation and evaporation by nanoparticles like mesoporous silica, can increase their antibacterial activities. Using nanoparticles, especially silver, is a suitable strategy for controlling *P. syringae*. However, high concentrations of silver nanoparticles are toxic. Bacteriophages and AMPs are recommended as alternatives to control bacterial infections in agriculture, including *P. syringae*. Combined treatments of phages and secondary metabolites have shown higher efficacy, potentially overcoming resistance. However, bacteriophages and AMPs are expensive and limited. In the end, using secondary metabolites and nanoparticles at low concentrations presents economic benefits and antibacterial activities without phytotoxic properties.

## INTRODUCTION

*Pseudomonas syringae *is a gram-negative microorganism responsible for various diseases in plants, including some species of fruits, grains, and flowers causing diseases like speckling, spots, and blight [1, 2]. *P. syringae* has two organized phases of growth: the epiphytic phase, when it lives on the external portions of plant tissues (usually aboveground), and the endophytic phase, when bacteria enter the plant tissue and take over the intercellular apoplast space [3]. The lesions formed in hosts are related to virulence factors controlled by quorum sensing [4]. The pathogenicity of this bacterium is due to biofilm formation, toxins, hormones, and enzymes that degrade cell walls [5]. Some protective strategies against *P. syringae* include a balanced nutrient supply, drip irrigation, sanitizing pruning tools, removing symptomatic plants, and planting healthy plants. However, the rapid spread of the bacterium makes it difficult to control the disease [6].

Several antimicrobial compounds can control this disease, such as chemical compounds, biological agents, secondary metabolites, nanoparticles, bacteriophages, and AMPs. The recent chemical treatment of *P. syringae* is highly dependent on spraying bactericidal compounds like copper formulations and streptomycin. The best available prevention of *P. syringae* is chemical control in the early phase of the disease [7]. Using copper compounds as antimicrobial agents has been used to decrease symptoms of canker disease for more than a century. However, other control methods must be replaced because of environmental pollution and bactericide resistance among *P. syringae* [8]. Streptomycin has been used as an antibiotic to control *P. syringae* since the 1950s [9]. Controlling bacteria with antibiotics has its limitations and excessive use of antibiotics can lead to bacterial resistance [10]. Although there have been few successes in the biological control of bacterial pathogens in nature, biological control using antagonistic bacteria is another strategy to control *P. syringae* [11-15].

Designing novel methods to decrease damages caused by these bacterial pathogens is essential. New studies focus on secondary metabolites and their applications to control plant microbial pathogens [16]. Researchers have studied the antibiotic effects of the main constituents of different aromatic plants against a comprehensive range of microorganisms including *P. syringae* [17]. Although essential oils are potential antimicrobials, their inherent features like volatility in aqueous environments and hydrophobicity make them less effective. To increase the antimicrobial effects of essential oils and their resistance to evaporation or decomposition, researchers suggested using mesoporous silica nanoparticles [18]. Using nanoparticles is a known strategy for controlling *P. syringae*. Ions and silver nanoparticles (AgNPs) are widely used for different purposes, including protective factors for plants and antimicrobial agents that are safer than artificial pesticides [19]. Scientists reported that combining chitosan with silver nanoparticles could promote the antimicrobial effect against *P. syringae* in vitro [20]. Bacteriophages can kill their specific host bacteria and are not toxic; they are self-replicating and therefore are better-controlling agents than antibiotics against phytopathogens [21]. Phages such as PN05 and PN09 offer a promising alternative for *P. syringae* control. The appearance of mutants resistant to phages limits their efficacy. Combination therapies can overcome bacterial resistance [22]. The emergence of resistant pathogens to conventional antibiotics forced researchers to find new antimicrobial agents from natural sources. AMPs such as battalions is another antimicrobial agent to control bacterial canker [23, 24]. Antimicrobial peptides interfere with the metabolism to exert their antimicrobial against bacterial pathogens [25]. AMPs, including synthetic and natural types, are popular because they control diseases in humans and plants instead of antibiotics [26]. This study aims to investigate different methods to control cankers and to find the best method.

**Chemical Compounds**

Using bactericides is the most popular method for managing diseases caused by *P. syringae*. These chemical compounds primarily include various forms of copper, such as the "Bordeaux mixture," (copper sulfate), cupric hydroxide, copper salts of fatty acids, and ammoniacal copper, as well as other heavy metals [27, 28]. Chemical control can effectively prevent the disease in the initial stage [7]. 

Copper compounds serve as effective antimicrobial agents against plant diseases [29]. Recently, the use of antibacterial compounds containing copper to combat *P. syringae* has gained attention [7]. Copper, with its three oxidation states and its role in enzymes, is involved in redox reactions in both prokaryotes and eukaryotes. However, additional amounts are toxic to plant pathogenic bacteria [30]. In oxic and anoxic situations, copper can damage proteins and infiltrate the iron-sulfur centers of multiple proteins, respectively [31]. Researchers have shown that the minimum inhibitory concentration (MIC) of copper sulfate in *P. syringae* strains ranges from 0.75 to 3.0 mM (Table 1) [32]. 

**Table 1:** Summary of several strategies to control canker plant disease caused by *P. syringae*

**Antimicrobial Compound**		**Example**	**MIC**	**Mechanism**	**Challenges**	**Ref.**
**Chemical compounds**		Streptomycin	300 - 900 μg·m l^–1^	Bonding to ribosomal rRNA (part 16SrRNA from 30SrRNA) of bacteria and interfering on translation of mRNA causing cell death	Emergence of resistant pathogens Changing communities of bacteria Environmental pollution	[32] [37]
		Copper sulphate	0.75-3.0 mM	Penetration iron sulfur centers of proteins in the cells which are in anoxic situations Causing lipid peroxidation with catalysis of a Fenton-like reaction		[31] [32]
**Biological control**		*B. subtilis* *P. agglomerans* *L. plantarum*	- - -	Creation of toxins, hydrolases, lipopeptides, and extracellular antibiotics Nutritional competition and production of organic acids with antibacterial effects and peptide antibiotics Production of inhibitory bioactive composites, bacteriocins and organic acids		
					Presentation of few successes to control of bacterial pathogens in nature	[42] [43] [44] [45] [48] [49]
**Secondary metabolite of plants**	Phenols	*Thymus- Rosmarinus- Foeniculum- Mentha*	3.92-125 mg ·m l^–^	The disruption of cell wall Inhibition of efflux pumps Disturbances in ATP balance Alteration in protein synthesis	High volatility Rapid degradation	[57] [59]
	Terpenoids	*Oregano- Rosmarinus*				
**Nanoparticles**		AgNPs	12 ppm	Changing structure by penetration to the cell wall of bacteria Formation of free radicals by the silver nanoparticles Interaction with the thiol groups of many vital enzymes	Nanotoxicity of silver	[65] [66] [67] [70]
**Bacteriophages**		PN05 and PN09	2 mg ·m l^–^	Attachment and insertion of their genetic contents into the host procaryote cells Controlling machinery of hosts Replication of their nucleic acids Packaging	Creating mutants resistant to phages	[21] [76] [77]
**Antimicrobial peptides:** **Antibacterial Synthetic Analogues of Battacin**	synthetic	Cyclic	5- 98 μM	Perturbing the phospholipid Interfering with metabolism Targeting cytoplasmic components	Identification Purification methods	[96]
		linear	1− 24 μM			
	Natural	Battacin	5−10 μM			

Pathogenic strains of *P. syringae* that have long-term contact with copper bactericides express a periplasmic protein (CopA) that can bind to copper and confer copper resistance. The expression of copB produces an outer membrane protein expected to play a role in binding copper to copA [33]. Frequently spraying with copper bactericides also leads to the emergence of copR and copS genes in the *P. syringae* strains, resulting in maximum copper resistance. All copper-resistant strains contained at least one of two plasmids, pPaCul (about 70.5 kb) or pPaCu2 (about 280 kb), or both. In a copper-resistant strain, Pa429, the location of the copper-resistance gene(s) was studied by insertional inactivation with Tn*5*. The MIC of copper sulfate in the copper-sensitive mutant obtained by Tn*5* tagging reduced from 2.75 to 0.75 mM. The 14.5 kb *Bam*HI fragment designated pPaCuB14 containing the same locus mutagenized with Tn*5* was cloned from pPaCu1. However, pPaCuB14 did not confer copper resistance in the transformant of copper-sensitive strain Pa21R, suggesting that this clone did not contain a full set of copper-resistance gene(s). In this study, a cosmid library of pPaCu1 was made, and six cosmids, clones hybridized with pPaCuB14 were selected. One of the six cosmids, designated pPaCuC1, conferred a near wild-type level of copper resistance in the transformant of the copper-sensitive strain. pPaCuC1 had a homologous region that hybridized with all of the PCR-amplified fragments of *copA, copB, copR, *and *copS *genes of *P. syringae *pv. *tomato. *DNA sequence analysis of the homologous region revealed the existence of four open reading frames (ORF A, B, R, and S) oriented in the same direction. The predicted amino acid sequences of ORFs A, B, R, and S, had 80, 70, 97, and 95% identity with CopA, B, R, and S of *P. syringae *pv. *tomato, *respectively. [32] .When excessive copper compounds are used as bactericidal sprays, they can be toxic to the plant, causing discoloration in the stem and spots on the underside of leaves [9]. Soil pollution with copper compounds causes severe environmental difficulties, particularly toxic effects on plants, animals, and humans [34]. Excessive accumulation of copper in the tissue in Wilson's disease causes neurological dysfunction and progressive cirrhosis [35]. Although the application of copper is a common method against *P. syringae*, the problems caused by it, including environmental pollution and resistance to this bactericide, have necessitated the search for alternative methods [8].

Another chemical control for *P. syringae* is the aminoglycoside antibiotic streptomycin [36]. It has been used to control phytopathogens since the 1950s and is considered the most significant active current chemical treatment for *P. syringae* [9]. Streptomycin attaches to the small subunit of the procaryote ribosome, causing cell dysfunction and death [37]. Controlling bacteria with antibiotics has its limitations. Excessive use leads to resistance of pathogens in microbial communities [10]. Frequent use of antibiotics has caused resistance in plant pathogens, including *P. syringae*, which is attributed to the presence of pPaCu1 and pPaCu2 plasmids. Studies have shown that the MICs increased from 300 to 900 μg·m l^–1^ (Table 1) [32]. Streptomycin resistance is due to three main mechanisms: altering the structure of streptomycin, changing its Ribosomal Binding Site (RBS), and decreasing streptomycin uptake. The first two mechanisms are utilized by *P. syringae* strains [38]. Unfortunately, these two chemicals do not work well due to their toxicity and bacterial resistance.

**Biological Control**

Environmental concerns have focused on advancing biological control agents as a substitute, environmentally friendly approach for protecting horticultural and agricultural yields against phytopathogens [39]. Biological control is an alternative strategy to control *P. syringae*. In recent decades, several bacteria like *Lactobacillus plantarum*, *Bacillus subtilis, *and* Pantoea agglomerans* have been used for this purpose [11, 13, 15, 40]. 

*B. subtilis* has long been used as a biological control agent against phytopathogens [41]. Bacillus species have antagonistic effects by producing toxins, antibiotics, hydrolases, and lipopeptides. Their metabolites have a broad ability and are also safe for humans [42]. The significant effect of treatment with Bacillus strains is the control of diseases caused by *P. syringae* [11]. 

*P. agglomerans* species can control bacterial diseases that occur after harvests, such as basal kernel blight of barley caused by *P. syringae* or fire blight caused by *Erwinia amylovora* [15, 40]. *P. agglomerans* fight with phytopathogens through nutrient competition and the production of antimicrobial compounds, organic acids, and peptides [43-45]. The strain SWg2 of *P. agglomeran*s (GenBank, KC783460) is an antagonistic endophytic bacterium against *P. syringae pv. Mori,* isolated from the roots of healthy blackberry plants [46]. It has been reported that this bacterium can produce various antibiotics such as Herbicolin I (APV), Pantocin A (Herbicolin O), Pantocin B, Andrimd, and AGA (alanylgriseoluteic acid) [47]. 

Lactic acid bacteria (LAB) are good candidates for developing microbial biopesticides. *L. plantarum* is a suitable lactic acid bacterium that produces metabolites like organic acids and bacteriocins, which can eliminate pathogens [48, 49]. Biocontrol strategies have shown promising results against *P. syringae* in vitro. However, there have been few successes in the biological control of bacterial pathogens in vivo [13, 50].

**Secondary Metabolites of Plants**

Several papers have been published about the antimicrobial activity of secondary metabolites, such as essential oils [51]. Essential oils of plants mainly consist of two important chemicals: terpenoids with various carbon skeleton and oxygenated derivatives, and phenylpropanoids [52]. Polyphenols and terpenoids are plants' secondary metabolites with antibacterial activities, composed of Phenylpropanoids with one or more C6-C3 units [53]. Essential oils are composed of volatile and semi-volatile compounds extracted by different distillations or mechanical methods [54].

Essential oils have defensive mechanisms against phytopathogens; however, they are not involved in plant growth [55]. The main goal of using essential oils in agriculture is to decrease chemical usage [56]. They use different mechanisms, including the destruction of the cell wall and membrane of pathogens, causing the cytoplasmic contents to leak out, disrupting the balance on both sides of the membrane, and finally leading to cell lysis and death [57].

Various essential oils are described as having antimicrobial activity against pseudomonas  [58]. Scientists studied the antimicrobial activities of essential oils from six plants, namely *Rosmarinus officinalis, Thymus diagenesis, Foeniculum vulgare, Mentha spicata, Mentha piperita*, and *Pelargonium graveolens *against two *P. syringae* strains. The antimicrobial activities of the oils were tested in the present study, and the MIC values evaluated their influences. The study of the MIC of essential oils on the studied strains indicated that all the strains were sensitive, with values ranging from 3.92 to 125 mg · ml^–1^. However, the essential oil of *T. daenensis* had the most antimicrobial effect and the lowest MIC (3.92 μg·m l^–1^) against *P. syringae* strains IVIA 773-1, which is related to thymol. In contrast, the essential oil of *R. officinalis* had the lowest inhibitory effect on the plant pathogen *P. syringae* strains W1 (Table 1) [59]. Volatility and rapid degradation are two features of essential oils that restrict their usage. Researchers used mesoporous silica nanoparticles (MSNPs) to increase the shelf life of essential oils and prevent their evaporation and degradation, thereby enhancing their antibacterial properties and controlling their release rate. Two of the most effective essential oils, extracted from* cinnamon* (*Cinnamomum zeylanicum*) and *mustard* (*Brassica nigra*) at a concentration of 0.016% (v/v), had bacteriostatic effects after 24 h. The encapsulation of essential oils into MSNPs, compared with free essential oil, increases their potency of antimicrobial effect tenfold. Cinnamaldehyde in MSNPs can decrease *P. syringae* growth by more than 99.9% and can treat and stop pathogenesis in yields, enabling more control of volatile compounds (Table 2) [18]. Results suggest that the essential oils can be used as antibacterial agents against phytopathogens. However, more studies are needed to obtain an economical combination without any toxic effects on the host plant.

**Nanoparticles**

Using nanoparticles is a well-known strategy for controlling *P. syringae*. Recently, inorganic nano-biocides, such as silver, titanium dioxide, and zinc oxide, have received excessive attention for plant disease management. Silver-based antibacterial agents are particularly considered due to their excellent antibacterial action [60, 61]. Silver ions and nanoparticles AgNPs have antimicrobial effects, and one of their applications is protecting plants against phytopathogens [19]. Silver’s features have been known in medicine for more than 2,000 years, and it has been used ever since. The toxicity of Silver-based compounds on main species of microorganisms has caused their use as antimicrobial agents since the nineteenth century [62, 63]. AgNPs have the potential for antimicrobial activity against bacterial and fungal phytopathogens. AgNPs show antimicrobial activity against* Erwinia sp., P. syringe, B. megaterium, F. graminearum, F. avenaceum, and F. culmorum* [64]. 

So far, various mechanisms have been proposed for the function of nanoparticles as antimicrobial compounds, including accumulation on the cell membrane and creating a hole in it, leading to the death of the microorganism, but these mechanisms are still debated [65]. Another mechanism predicted for the function of nanoparticles against bacteria is the creation of free radicals by nanoparticles, which can cause cell lysis and ultimately death by creating pores on the surface of the microorganism's cell [66]. Silver ions can connect with the thiol groups of many vital enzymes and disable them [67]. DNA consists of phosphate units, and in another mechanism, nanoparticles cause the death of microorganisms by affecting bases and destroying them [68]. In another mechanism, nanoparticles cause changes in the phosphotyrosine of bacterial peptides and phosphorylate them, stopping cell growth and resulting in cell death [69,70]. Researchers investigated the practical bactericidal effect of silver nanoparticles against *P. syringae*. In that research, in vitro assessment showed that the MIC concentration of AgNPs against *P. syringae* strain 21 was 12 ppm (Table 1). The type and concentration of nanoparticles determine their effectiveness as antibiotics against *P. syringae*. 

Chitosan is a linear polysaccharide commonly used with silver to control plant bacteria, having antimicrobial effects against phytopathogens [71]. Chitosan and its derivatives, with their positively charged amines, can contact negatively charged proteins on the cell surface of bacteria and create pores on them, causing cytoplasmic elements to outflow and result in cell death. [72]. Scientists combined chitosan, with silver nanoparticles and investigated their antibacterial effects against canker disease in plants caused by *P. syringae*. The results of this study showed that AgNPs in combination with chitosan (MIC 12 ppm) had more antibacterial properties than single AgNPs (MIC 4-9.2 ppm) (Table 2) [70]. The association of chitosan with silver nanoparticles increases their antibiotic effects in vitro. Despite the effectiveness of silver nanoparticles and their antimicrobial effects on pathogens, high concentrations can be extremely toxic and dangerous, stressing the need for more research [73].

**Table 2:** Summary of several antimicrobial compounds to control canker plant disease caused by *P. syringae*

**Antimicrobial Compound**	**Result**	**Description**	**References**
Essential oils: Cinnamaldehyde into mesoporous silica nanoparticles (MSNPs)	Eliminating more than 99.9% of bacterial growth of *P. syringae*	Increasing the stabilization of compounds Extending and improving their antimicrobial	[18]
AgNPs/chitosan nanocomposite	MIC 4-9.2 PPM	Improving of the antimicrobial property of silver nanoparticles	[70]
applications of bacteriophages and carvacrol together	Phages and carvacrol 2 mg ·m l^–1^	Overcoming resistance development	[22]

**Bacteriophages**

Bacteriophages are suggested as a substitute to control phytopathogens, including *P. syringae*. Phages are bacterial viruses that have specific hosts and intense lytic action [74]. Once they enter their host, they enter one of two life cycles: The lytic life cycle or the lysogenic life cycle [75]. In the first step, phages attach to the host cell and then inject their genetic content into it [76]. After inserting their genetic content into the host cell, if they enter in lytic cycle, the phages can replicate their nucleic acid contents and then form new bacteriophage particles [77].

Their nature to have a specific host and their self-replication and non-toxicity make bacteriophages better than antibiotics [21]. Bacteriophages are the most numerous organisms in the world, and the balance of microbes throughout the world depends on them [78]. They are present in a wide range of bacterial hosts in different habitats, and they have different sizes. For example, T4 phages are the largest, with a length is about 0.2 µm with their diameter of about 0.08-0.1 µm [79]. The majority of phages consist of tails through which their genetic content is transferred to their host [80]. 

Phages also have commercial applications. For example in agriculture, they can be used as Agri phages by OmniLytics to control the tomato pathogen *P. syringae* pv [81]. Numerous bacteriophages against different pathovars of *P. syringae* have been studied [82]. Scientists reported the practical bactericidal effect of phages PN05 and PN09 against *P. syringae*. PN05 and PN09 are phages with double-stranded DNA that belong to the family Myoviridae. In that study, in vitro evaluation disclosed that MICs of these phages against *P. syringae* were 2.0 mg ·m l^–1^, preventing *P. syringae* growth (Table 1). Phages such as PN05 and PN09 showed a hopeful substitute for *P. syringae* control. However, with the appearance of mutants that are resistant to phages, their efficacy decreased. Additionally, bacteriophage and carvacrol have been studied to overcome resistance, which may be valuable for controlling *P. syringae*. The combined treatment of phages and carvacrol can be used to overcome resistance progress. The results showed that the combined treatment (2.0 mg ·m l^–1^), is more efficient against *P. syringae* growth. Using phages and carvacrol at 2.0 mg ·m l^–1 ^could successfully decrease the growth of *P. syringae* (Table 2). Combining carvacrol and phages can decrease biofilm growth and eliminate pre-formed *P. syringae* biofilms. Therefore, phage therapy may be a possible way of controlling *P. syringae*, and its efficacy increases when combined with the natural antimicrobial carvacrol [22]. 

**AMPs**

MPs are considered as alternatives to previous antibiotics against plant pathogens [26]. AMPs are small-molecule ribosomal or non-ribosomal polypeptides generated from the cleavage of larger protein segments and are modified with further post-translations [83, 84]. Many AMPs are antimicrobics with wide effects against pathogenic procaryotes (bacteria), eucaryotes (fungi and parasites), and viruses. [85]. These peptides are members of innate immunity that operate against pathogens as parts of the first line of defense in humans and other higher organisms [86, 87]. AMPs are effective on single-cell microorganisms with similar nutritional needs (88). AMPs have some similar characteristics, like positive charges, hydrophilic and hydrophobic areas, and short chains of amino acids with 12-50 residues, but they have diversities between species (89), and the identification of their sequences is increasing [90, 91].

The mechanisms of action of AMPs have been examined by various biological, biochemical, and biophysical methods [92]. AMPs attack the plasma membrane and cell components and at the same time have no harmful effects on plants [93, 94]. Microorganisms don`t gain resistance to AMPs; moreover, they can destroy multidrug-resistant microorganisms at low concentrations [95]. AMPs are effective molecules that fight against bacterial infection with different mechanisms, like the destruction of the cell membrane bilayer, cytoplasmic components, and disturbance metabolism [96]. AMPs have been isolated from insects, amphibians like frogs, and phagocytic vacuoles of mammals [97]. 

Antibiotic resistance has worried healthcare professionals and persuaded them to seek substitute therapies [98]. Since AMPs have less resistance to microorganisms compared to other antimicrobial agents, they have recently received more attention [99, 100]. Placing them in nanostructures and their unconventional structure can solve the problem of their susceptibility to protease degradation [101, 102]. Various AMPs are known as components with high bactericidal activity against phytopathogens [103]. Plant Pathogenic *Pseudomonas strains *have generated attention as a target for new treatments based on AMPs [104, 105]. Different concentrations of AMPs can disrupt the *P. syringae* membrane [106]. One of the most tested AMPs is BP100, which has been confirmed as an antibacterial agent on phytopathogens [107]. The antibacterial effect of this AMP is established against *E. amylovora, Xyllela fastidiosa, *and* Dickeya chrysanthemi *and tested against *P. syringae*. [108-111]. 

Researchers produced the first synthetic lipopeptide with antibacterial and antibiofilm activity, battacin. The mechanism of action of this lipopeptide was the lysing membrane of microorganisms. In contrast to natural ribosomal AMPs, lipopeptides are non-ribosomal antibiotics with N-terminal conjugated to fatty acids with long chains, and different structures that treat methicillin-resistant *S. aureus* (MRSA) infections [112, 113]. The appearance of resistance to polymyxin B, which is the last line of defense for serious infections, and its nephrotoxicity highlight the need for the production of short peptides with broad-spectrum antimicrobial activities. Battacin, a new cyclic lipopeptide, is isolated from *Paneibacillus tianmunesis.* It belongs to peptide antibiotics with the octapeptin group, which contains a high percentage of α γ diaminobutyric acid (Dab) and other nonprotein amino acids, and also features a branched fatty acid tail with β-hydroxy, connected to a cyclic heptapeptide moiety [23, 24]. Battacin is described as a better antibiotic than polymyxin B. In vitro assessment showed that MICs of battacin and synthetic analogs of battacin, including cyclic and linear forms, against *P. syringae* were 5-10, 5-98, and 1-24 µm, respectively (Table 1). Linear lipopeptides are more powerful and economical antibiotics than their cyclic counterparts [114]. The synthesis of AMP is expensive; however, they are recognized to be effective and can become beneficial in managing *P. syringae*.
